# Dose-dependent positive association between cigarette smoking, abdominal obesity and body fat: cross-sectional data from a population-based survey

**DOI:** 10.1186/1471-2458-11-23

**Published:** 2011-01-11

**Authors:** Carole Clair, Arnaud Chiolero, David Faeh, Jacques Cornuz, Pedro Marques-Vidal, Fred Paccaud, Vincent Mooser, Gérard Waeber, Peter Vollenweider

**Affiliations:** 1 Department of Ambulatory Care and Community Medicine, University of Lausanne, Lausanne, Switzerland; 2 Institute of Social and Preventive Medicine (IUMSP), University of Lausanne, Lausanne, Switzerland; 3 University Hospital Center (CHUV), University of Lausanne, Lausanne, Switzerland; 4 Cardiomet, University Hospital Center (CHUV), Lausanne, Switzerland; 5 Medical Genetics, GlaxoSmithKline, Philadelphia, Pennsylvania, USA

## Abstract

**Background:**

Although smokers tend to have a lower body-mass index than non-smokers, smoking may favour abdominal body fat accumulation. To our knowledge, no population-based studies have assessed the relationship between smoking and body fat composition. We assessed the association between cigarette smoking and waist circumference, body fat, and body-mass index.

**Methods:**

Height, weight, and waist circumference were measured among 6,123 Caucasians (ages 35-75) from a cross-sectional population-based study in Switzerland. Abdominal obesity was defined as waist circumference ≥102 cm for men and ≥88 cm for women. Body fat (percent total body weight) was measured by electrical bioimpedance. Age- and sex-specific body fat cut-offs were used to define excess body fat. Cigarettes smoked per day were assessed by self-administered questionnaire. Age-adjusted means and odds ratios were calculated using linear and logistic regression.

**Results:**

Current smokers (29% of men and 24% of women) had lower mean waist circumference, body fat percentage, and body-mass index compared with non-smokers. Age-adjusted mean waist circumference and body fat increased with cigarettes smoked per day among smokers. The association between cigarettes smoked per day and body-mass index was non-significant. Compared with light smokers, the adjusted odds ratio (OR) for abdominal obesity in men was 1.28 (0.78-2.10) for moderate smokers and 1.94 (1.15-3.27) for heavy smokers (*P *= 0.03 for trend), and 1.07 (0.72-1.58) and 2.15 (1.26-3.64) in female moderate and heavy smokers, respectively (*P *< 0.01 for trend). Compared with light smokers, the OR for excess body fat in men was 1.05 (95% CI: 0.58-1.92) for moderate smokers and 1.15 (0.60-2.20) for heavy smokers (*P *= 0.75 for trend) and 1.34 (0.89-2.00) and 2.11 (1.25-3.57), respectively in women (*P *= 0.07 for trend).

**Conclusion:**

Among smokers, cigarettes smoked per day were positively associated with central fat accumulation, particularly in women.

## Background

Recent studies suggest that smoking is associated with metabolic disorders such as diabetes and obesity [1,2]. Obesity is a risk factor for cardiovascular disease; in particular, the intraabdominal accumulation of body fat (BF) may confer a higher risk of developing diabetes [3], cardiovascular disease [4] and death [5], independently of general obesity. The co-existence of obesity and smoking is expected to occur more frequently [6,7]. Persons with both conditions are at high risk for cardiovascular disease and cancer, and have a substantially reduced life expectancy [5,8]. Thus, it is crucial to better understand the effects of smoking on obesity and its associated conditions.

The association between smoking and obesity is complex. On one hand, smokers have a lower body weight and body-mass index (BMI) than non-smokers [9]. On the other hand, current smokers tend to have a larger waist circumference (WC) and a higher waist-to-hip ratio than non-smokers, suggesting that smoking may favor the accumulation of abdominal fat [10-14]. In addition, among smokers, the number of cigarettes smoked seems to be directly associated with WC and BMI [10,14-17]. BF, measured by bioimpedance, is another marker of obesity. To our knowledge, the association between smoking and the amount of BF has not been previously assessed in a large general population.

In this study, our objective was to assess the association between the number of cigarettes smoked per day and measured WC, BF, and BMI in a large population-based study. Our hypothesis was that heavy smokers (who smoke more than 20 cigarettes per day) have higher WC, BF, and BMI compared with light smokers.

## Methods

### Study sample

**Figure 1:**
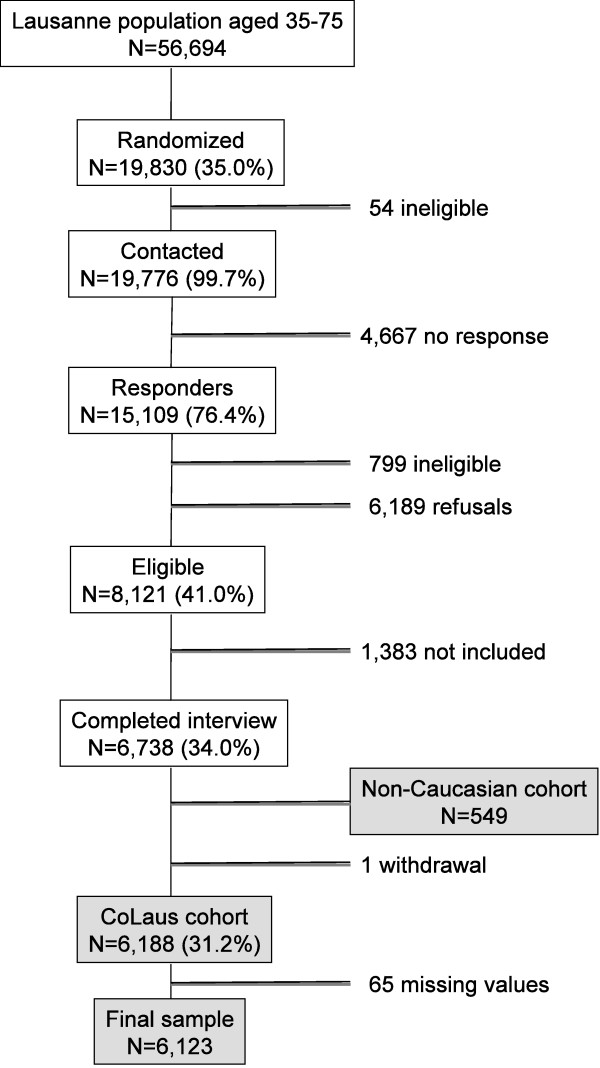
**Sampling procedure**.

We analyzed the baseline data from the CoLaus study, a cross-sectional, population-based study of 6,123 participants. The details of the CoLaus study have been previously described in detail [18,19]. Briefly, the CoLaus Study was designed to investigate the prevalence and genetic determinants of risk factors for cardiovascular disease. The survey started in 2003 and was approved by the Institutional Ethics Committee of the University of Lausanne. All subjects between 35 and 75 years of age living in Lausanne (Switzerland) were identified from a city register, and a random sample of 19,830 subjects (35% of the overall population) was invited to participate by mail. Inclusion criteria included providing written informed consent, being 35-75 years of age, and of Caucasian origin. The last inclusion criterion was chosen for the genetic arm and analysis of the study. Of the initial 19,830 subjects sampled, 54 subjects were considered as non-eligible before contact and 15,109 (76%) responses were obtained. A total of 4,667 subjects did not respond. Among responders, 6,189 (41%) subjects refused to participate in the study and 799 (5%) were considered as non-eligible. The sample of 8,121 subjects who agreed to participate represented 41% of the initially sampled population, 54% of all responders and 57% of all eligible responders. As there were more eligible participants than requested for the initial study, the last 1,383 subjects were not included in the study; a further 549 non-Caucasians were also excluded, and one participant initially included withdrawn from the study. Further, for our analysis, we included only participants with complete data for the main variables of interest. We excluded an extra 65 participants because of missing values for BMI (n = 1), WC (n = 1), BF (n = 57), smoking status (n = 2), education (n = 5) and marital status (n = 2) (Figure 1). Subjects interested in participating were then contacted by telephone and sent the first questionnaire by mail, which recorded information on demographic data, socioeconomic status, and several lifestyle factors, namely tobacco and alcohol consumption. All eligible participants were then asked to attend an outpatient clinic in the morning after an overnight fast, and data were collected by trained field interviewers during a single visit. The first questionnaire mailed with the appointment letter and completed by the participant prior the visit was reviewed. A second questionnaire, focused on personal and family history of disease as well as cardio-vascular risk factors and treatment, was given during the interview. Trained nurses measured body height, weight, waist and hip circumferences and BF during this visit.

### Clinical data

Data on smoking included the subject's previous and current smoking status as well as the amount of tobacco smoked and age at which the subject began smoking, and, if they were former smokers, when they stopped smoking. Participants were categorized as non-smokers if they had never smoked, former smokers if they had quit smoking at the time of the interview, as current smokers if they were currently smoking ≥1 cigarette per day, and as "other smokers" if they were currently smoking pipe, cigars, or cigarillos. Based on current smoking categories, current smokers were divided into 3 predefined categories according to daily consumption; light (1 to 9 cigarettes/day), moderate (10 to 19 cigarettes/day), and heavy smokers (≥20 cigarettes/day). For light smokers, most recent studies used a cutoff of 10 cigarettes/day [20,21]. We considered people smoking ≥ 20 cigarettes/day as heavy smokers because this corresponds to the quantity of cigarettes contained in a standard pack in Western countries and other studies have also used this cutoff [21,22]. Data on alcohol consumption was collected, including past and current drinking habits as well as the amount of alcoholic beverage units consumed the week prior to the interview. At-risk alcohol consumption was defined as an intake of more than 14 drinks/week for men <65 years of age, and more than 7 drinks/week for women all ages or men ≥65 years of age [23]. Body weight and height were measured with the participants standing barefooted in light indoor clothing. Body weight was measured in kilograms to the nearest 100 g. Height was measured to the nearest 5 mm. BMI, defined as weight/height^2^, was calculated and subjects were classified as underweight (BMI <18.5 kg/m^2^), normal (BMI ≥18.5 and <25 kg/m^2^), overweight (BMI ≥25 and <30 kg/m^2^), or obese (BMI ≥30 kg/m^2^) according to WHO criteria [24]. Waist and hip circumferences were measured with a non-stretchable tape. The waist was measured over the abdomen but under the clothing at a level midway between the lower rib margin and the iliac crest, and the measurement was rounded to the nearest centimetre. Two measurements were taken and the mean was used for analyses. Abdominal obesity was defined according to the literature (Table 1) [25]. BF and fat-free mass were assessed by electrical bioimpedance using the Bodystat^® ^1500 body mass analyser (Bodystat Ltd, Isle of Man, England) [26]. Subjects had to fast for at least 8 hours, not engage in strenuous physical activity during the previous 12 hours, and abstain from consuming caffeine or alcohol-containing beverages for 24 hours before the examination. All metallic objects were removed from the body and clothing, and the measurement was performed after a 10-minute rest in the supine position. BF was expressed as a percentage of total body weight. Excess BF was defined if BF mass (expressed in percent) was superior or equal to the 95^th ^percentile for the Swiss population according to Kyle et al [27] (Table 1).

**Table 1:** Thresholds used to define waist circumference (WC) categories [28] and to define excess of body fat (BF) mass [30].

	Men	Women
Waist (cm)		
Normal	<94	<80
Medium	≥94 and <102	≥80 and <88
Large	≥102	≥88
Body fat (%)*		
32-44 years	≥28.1	≥35.9
45-54 years	≥28.7	≥36.5
55-64 years	≥30.6	≥40.5
65-75 years	≥32.6	≥44.4

* These cutoffs correspond to BF mass ≥95^th ^percentile according to Kyle et al.[30]

### Statistical analyses

Statistical analyses were conducted using Stata version 10.0 (StataCorp, College Station, Texas). Age-adjusted mean WC, BF, and BMI were calculated for non-smokers, current smokers, and former smokers using linear regression models. Current smokers were stratified according to the number of cigarettes smoked per day. The age-adjusted and multi-adjusted (adjusted for age continuous, education level categories and alcohol consumption categories) odds ratios (ORs) between WC and BMI categories and smoking categories (expressed in cigarettes per day) were estimated by applying a maximum-likelihood multinomial (polytomous) logistic regression model for men and women separately. For BF, we applied a logistic regression model for men and women separately. Uni- and multivariable multinomial logistic and logistic regression models were fitted for smokers only. We decided to adjust for education level (using predefined 5 levels categories) and alcohol consumption (comparing those at risk, defined as having an intake of more than 14 drinks/week for men <65 years of age, and more than 7 drinks/week for women of all ages or men ≥65 years of age, versus those not at risk), because these factors may confound the association between smoking and outcome. We chose not to adjust WC for BMI or vice-versa to avoid over adjustment, because there was a strong correlation between these variables. Statistical significance was considered as *P *< 0.05.

## Results

### Baseline characteristics

The baseline characteristics of the 6,123 participants (3,211 women and 2,912 men) are shown in Table 2. About a third of the men and a quarter of the women were current smokers. Men were more frequently obese or overweight than women, while the prevalence of abdominal obesity and excess BF was higher in women. Smoking and obesity were more frequent among less educated participants (data not shown).

**Table 2:** Baseline characteristics of participants.

	Male	Female	Both sexes
N	2912	3211	6123

Mean age, year (SD)	52.6 (10.8)	53.5 (10.7)	53.1 (10.8)

Mean BMI, kg/m2 (SD)	26.6 (4.1)	25.1 (4.9)	25.8 (4.6)

BMI categories, No (%)			

Underweight (BMI < 18.5 kg/m^2^)	21 (0.7)	84 (2.6)	105 (1.7)

Normal weight (BMI 18.5-24.9 kg/m^2^)	1073 (36.9)	1756 (54.7)	2829 (46.2)

Overweight (BMI 25-29.9 kg/m^2^)	1325 (45.5)	909 (28.3)	2234 (36.5)

Obesity (BMI ≥ 30 kg/m^2^)	493 (16.9)	462 (14.4)	955 (15.6)

Mean waist circumference, cm (SD)	95.8 (11.2)	83.4 (12.5)	89.3 (13.4)

Waist categories, No (%)			

Normal waist	1297 (44.5)	1413 (44.0)	2710 (44.3)

Medium waist	840 (28.9)	734 (22.9)	1574 (25.7)

Large waist	775 (26.6)	1064 (33.1)	1839 (30.0)

Mean bodyfat* (SD)	23.8 (6.0)	34.3 (8.2)	29.3 (9.0)

Excess of body fat, No (%)			

No	2517 (86.4)	2355 (73.3)	4872 (79.6)

Yes	395 (13.6)	856 (26.7)	1251 (20.4)

Smoking status, No (%)			

Non-smokers	934 (32.1)	1513 (47.1)	2447 (40.0)

Former smoker	1122 (38.5)	894 (27.8)	2016 (32.9)

Current smokers	856 (29.4)	804 (24.0)	1660 (27.1)

No of cigarettes per day, No (%) (n = 1515)			

1-10 cig/d	201 (27.4)	336 (43.1)	537 (35.5)

11-20 cig/d	327 (44.5)	336 (43.1)	663 (43.8)

>20 cig/d	207 (28.2)	108 (13.9)	315 (20.8)

Pack years, No (%) (n = 1472)			

0-10 UPA	152 (21.4)	235 (30.9)	387 (26.3)

10-25 UPA	236 (33.2)	271 (35.6)	507 (34.4)

25-50 UPA	214 (30.1)	204 (26.8)	418 (28.4)

>50 UPA	109 (15.3)	51 (6.7)	160 (10.9)

Highest level of education done, No (%)			

Obligatory school	503 (17.3)	770 (24.0)	1273 (20.8)

Apprenticeship	1110 (38.1)	1159 (36.1)	2269 (37.1)

High school	248 (8.5)	387 (12.1)	635 (10.4)

Master's degree	410 (14.1)	406 (12.6)	816 (13.3)

University	641 (22.0)	489 (15.2)	1130 (18.5)

Marital status, No (%)			

Single	449 (15.4)	558 (17.4)	1007 (16.5)

Married	1946 (66.8)	1655 (51.5)	3601 (58.8)

Divorced	478 (16.4)	752 (23.4)	1230 (20.1)

Widowed	39 (1.3)	246 (7.7)	285 (4.7)

Alcohol consumption, No (%)			

Abstinent	461 (15.8)	1194 (37.2)	1655 (27)

Consumption not at risk	1876 (64.4)	1574 (49.0)	3450 (56.3)

At risk consumption	575 (19.8)	443 (13.8)	1018 (16.6)

* Body fat is expressed as % of total body weight

SD = standard deviation, BMI = body-mass index, No = number, cig = cigarettes

### Mean adjusted WC, BF, and BMI according to smoking status and gender

**Figure 2:**
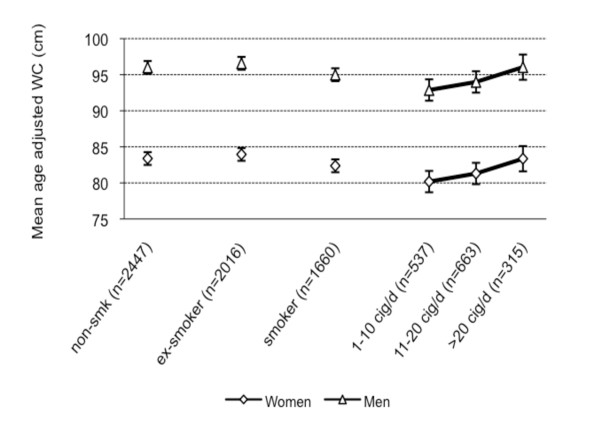
**Mean age-adjusted waist circumference and 95% Confidence Intervals by smoking status and gender**.

**Figure 3:**
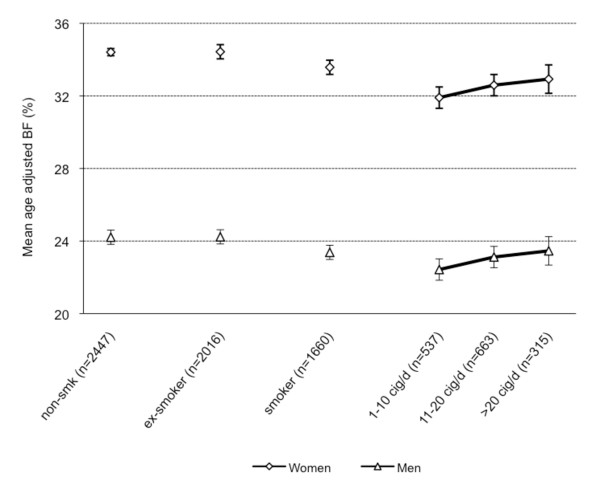
**Mean age-adjusted body-fat and 95% Confidence Intervals by smoking status and gender**.

**Figure 4:**
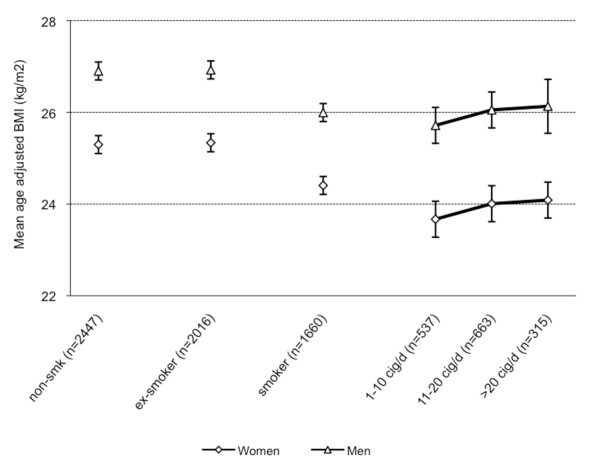
**Mean age-adjusted body mass index and 95% Confidence Intervals by smoking status and gender**.

Mean age-adjusted WC, BF, and BMI were calculated for non-smokers, current and former smokers. For current smokers, data were stratified according to number of cigarettes smoked per day (Figure 2, 3, 4). Non-smokers had a lower WC compared with former smokers but a higher WC compared with current smokers (Figure 2). Non-smokers had higher BF levels compared with smokers and about the same BF levels compared with former smokers. (Figure 3). Non-smokers had a higher BMI than current smokers and a lower BMI compared with former smokers (Figure 4). Multi-adjusted analyses (adjusted for alcohol consumption and educational level) were similar to the age-adjusted results (data not shown).

Among current smokers, gradients of higher WC, BF, and BMI levels with increasing numbers of cigarettes smoked per day were found. The trends were stronger for WC and BF than for BMI (Figure 2, 3,4). The results were similar in analyses using pack-years instead of number of cigarettes smoked per day (data not shown).

### Association between smoking and WC, BF, and BMI categories

Among current female smokers, heavy smoking was associated with increased odds of having abdominal obesity and excess BF compared with light smoking (Table 3). The results of the age-adjusted and multi-adjusted analyses were similar. Interestingly, we observed a trend such that the odds in either sex of having abdominal obesity increased with the number of cigarettes smoked daily. After adjustment for age, education level, and alcohol consumption this trend was statistically significant for abdominal obesity in both genders (*P *= 0.03 in men and *P *< 0.01 in women). Among men, we found that moderate and heavy smoking were associated with increased odds of having a medium WC compared with light smoking. No significant association between the number of cigarettes smoked daily and obesity (as defined as BMI) was found.

**Table 3:** Associations between waist circumference, body mass index and excess body fat categories and number of cigarettes among smoking men and women (n = 1515)

Men		Age-adjusted OR 95% CI		*P*	Multiple-adjusted* OR 95% CI		*P*
WC							
Medium waist	1-10 cig/d	1			1		
	11-20 cig/d	**1.70**	(1.10 - 2.63)	0.02	**1.73**	(1.11- 2.69)	0.02
	>20 cig/d	**1.83**	(1.13 - 2.97)	0.01	**1.82**	(1.12 - 2.96)	0.02
*P for trend*				*0.22*			*0.23*
Abdominal obesity	1-10 cig/d	1			1		
	11-20 cig/d	1.31	(0.80 - 2.14)	0.28	1.28	(0.78- 2.10)	0.32
	>20 cig/d	**1.93**	(1.15 - 3.25)	0.01	**1.94**	(1.15 - 3.27)	0.01
*P for trend*				*0.04*			*0.03*
BMI							
Overweight	1-10 cig/d	1			1		
	11-20 cig/d	1.26	(0.86 - 1.84)	0.23	1.22	(0.83 - 1.79)	0.31
	>20 cig/d	1.16	(0.76 - 1.76)	0.49	1.14	(0.75 - 1.74)	0.55
*P for trend*				*0.67*			*0.77*
Obesity	1-10 cig/d	1			1		
	11-20 cig/d	1.35	(0.75 - 2.40)	0.32	1.18	(0.65 - 2.12)	0.58
	>20 cig/d	1.31	(0.70 - 2.47)	0.39	1.21	(0.64 - 2.30)	0.55
*P for trend*				*0.98*			*0.91*
Body fat							
Excess of body fat	1-10 cig/d	1			1		
	11-20 cig/d	1.15	(0.64 - 2.09)	0.64	1.05	(0.58 - 1.92)	0.86
	>20 cig/d	1.18	(0.62 - 2.24)	0.62	1.15	(0.60 - 2.20)	0.68
*P for trend*				*0.95*			*0.75*

**Women**		**Age-adjusted OR 95% CI**		***P***	Multiple-adjusted* OR 95% CI		***P***

WC							
Medium waist	1-10 cig/d	1			1		
	11-20 cig/d	1.03	(0.71 - 1.51)	0.87	1.01	(0.69 - 1.48)	0.95
	>20 cig/d	1.20	(0.67 - 2.13)	0.55	1.17	(0.65 - 2.10)	0.61
*P for trend*				*0.02*			*0.04*
Abdominal obesity	1-10 cig/d	1			1		
	11-20 cig/d	1.15	(0.78 - 1.68)	0.49	1.07	(0.72 - 1.58)	0.74
	>20 cig/d	**2.40**	(1.44 - 4.02)	0.001	**2.15**	(1.26 - 3.64)	0.01
*P for trend*				*0.004*			*0.007*
BMI							
Overweight	1-10 cig/d	1			1		
	11-20 cig/d	1.33	(0.92 - 1.93)	0.13	1.27	(0.87 - 1.84)	0.22
	>20 cig/d	1.34	(0.79 - 2.27)	0.28	1.24	(0.72 - 2.13)	0.44
*P for trend*				*0.43*			*0.53*
Obesity	1-10 cig/d	1			1		
	11-20 cig/d	0.78	(0.45 - 1.35)	0.37	0.69	(0.39 - 1.22)	0.20
	>20 cig/d	1.46	(0.74- 2.89)	0.27	1.25	(0.62 - 2.52)	0.54
*P for trend*				*0.07*			*0.13*
Body fat							
Excess of body fat	1-10 cig/d	1			1		
	11-20 cig/d	1.42	(0.95 - 2.10)	0.08	1.34	(0.89 - 2.00)	0.16
	>20 cig/d	**2.29**	(1.38 - 3.81)	0.001	**2.11**	(1.25 - 3.57)	0.01
*P for trend*				*0.06*			*0.07*

* Statistical analyses by polytomous or logistic regression with adjustment for age, education, and alcohol consumption (at risk versus not at risk).

Results are expressed as odds-ratio (OR) with 95% confidence intervals (CI).

WC = waist circumference, BMI = body-mass index

## Discussion

In a middle-aged Swiss population, we found that among smokers of both sexes, WC increased with number of cigarettes smoked with some evidence of a linear trend suggesting a dose-response relationship. Among men, we observed that moderate and heavy smoking were associated with increased odds of having a medium WC compared with light smoking. Among women, heavy smoking was associated with significantly increased odds of excess BF compared with light smoking and the data suggested a trend though the p-values for trends did not reach statistical significance. While these findings are concordant with previous studies that showed a dose-dependent relationship between cigarette smoking and increased abdominal fat accumulation [11-14], this is the first study showing an association between the number of cigarettes smoked and total BF.

Various hypotheses may explain the counterintuitive finding of increased WC and BF among heavy smokers. First, nicotine *per se *could lead to fat accumulation. Indeed, several studies have shown that nicotine leads to insulin resistance [28,29], has an anti-estrogenic effect [30], and increases the level of stress hormones like cortisol [31]. Second, smokers are more likely to have unhealthy lifestyle habits, such as lack of physical activity, poor fruit and vegetable consumption, and increased alcohol consumption [32]. Such unhealthy behaviors favor weight gain and might partly explain why smokers tend to accumulate fat specifically in the abdominal area [1].Third, smokers tend to gain weight, especially fat mass, when they quit smoking [33]. Smokers usually make several attempts before they manage to quit smoking for an extended period, and even after they relapse they tend not to lose the weight they gained. This weight cycling after several previous attempts to quit smoking could explain why heavy smokers, who are more dependent on nicotine compared with light smokers, gain more weight.

Although the number of cigarettes smoked per day was associated with abdominal obesity in both sexes and medium WC in men only, the association between number of cigarettes smoked per day and BF was only significant in women. This is in accordance with studies reporting a stronger association between smoking and abdominal fat accumulation in women [11,12]. The sex difference could be explained by a stronger anti-estrogenic effect of nicotine in women than in men [30]. The fact that the association between numbers of cigarettes smoked per day and medium WC was not statistically significant among women (although there was a trend) might be due to the smaller sample size of women with medium WC.

In our study, smokers had on average lower BMI, WC and BF compared with non-smokers. This can be explained partly by the increased metabolism induced by nicotine [34]. However, among heavy smokers we found an inverse relationship, heavy smokers having a higher WC and BF compared with light smokers. The metabolic effects of nicotine that favor abdominal fat accumulation as well as the smokers propensity for unhealthy lifestyle habits that we mentioned previously might outweigh the increase in metabolism induced by nicotine among heavy smokers.

One of the study's strengths is the use of anthropometric measurements instead of self-reported weight and height. People tend to over report their height and under report their weight, resulting in an underestimation of BMI [35]. Under reporting of weight is more prevalent in those who are overweight or obese than in normal-weight persons [35]. Using self-reported data to determine BMI may lead to an overestimation of the association between obesity and any health condition [36]. This may explain why we found only a weak statistically non-significant association between elevated BMI and the number of cigarettes smoked daily, while a previous study using self-reported BMI did find a clear association [17]. We measured BF composition by bioimpedance. To our knowledge, no study has assessed the association between BF and smoking in a large sample population. In our study, 8% of men and 2% of women were considered obese according to BMI, but did not have excess BF according to the BF definition we used [27]. In contrast, 5% of men and 15% of women had excess BF, but were not considered to be obese according to their BMI. Our study suggests that heavy smokers may accumulate overall BF and abdominal fat (defined as high WC) without a corresponding increase in BMI.

This study has some limitations that should be acknowledged. First, the cross-sectional design of the study is a limitation. Direction of the causality between smoking and fat accumulation is disputable. Smoking might be used for weight control, especially by women [37]. Therefore, obese subjects may have increased the amount of cigarettes smoked to help with weight control.

Second, potentially confounding factors, such as physical activity or diet, were not assessed in our study. Poor diet, total energy intake as well as energy expenditure are associated with smoking and body size. They might therefore confound the relationship between smoking and anthropometric parameters. Adjustments for education level and alcohol consumption did not change the results. Nevertheless, a causal inference between smoking and fat distribution cannot be inferred based on our findings.

Third, the CoLaus study included only Caucasians. Therefore, the results may not necessarily generalize to other ethnic groups.

Fourth, the relatively low participation rate (41%) might also limit generalizability of our findings. Low participation rates are typical of surveys in Western countries, and our response rate was similar to that of the MONICA surveys conducted in Switzerland and other countries [38]. The magnitude of the non-participation bias is not proportional to the percentage of non-participants [39]. It is, however, possible that a disproportionate number of subjects who have both abdominal obesity and who were heavy smokers did not participate. If true, our findings would underestimate the strength of the associations.

Fifth, in our survey, former smoking was not defined according to time since quitting (smokers who have quit smoking for 6 months or more) as suggested by the literature [40], therefore, this category might include smokers who recently quit and in whom metabolic changes might not have occurred yet.

Finally, the validity of body fat mass depends on equations used to translate resistance and reactance measured with bioimpedance into BF and on data used as references to determine cutoffs. There is no consensus on which equations to use, and thresholds vary depending on the characteristics of the population used as a reference group (age, ethnicity, weight). The reference we used seemed appropriate for our sample because it was determined in a population similar to ours and the method was validated for older (>65 years) and obese subjects [41].

## Conclusions

Among smokers, the number of cigarettes smoked per day was positively associated with WC in men and women and with BF in women. The cross sectional design of our study and other limitations precludes to infer a causal relationship between smoking and fat distribution. However, current smokers should be informed that they are more prone to central fat accumulation and to the inherent additional health risks.

## List of abbreviations used

BF: body fat; BMI: body-mass index; WC: waist circumference; ORs: odds ratios;

## Competing interests

### Financial support

The CoLaus study was supported by unrestricted grants from GlaxoSmithKline and from the Faculty of Biology and Medicine of Lausanne, Switzerland, and is currently supported by a grant from the Swiss National Science Foundation 33CSCO-122661.

### Conflict of interest

None reported. Vincent Mooser is a full time employee of GlaxoSmithKline.

## Authors' contributions

CC has leaded the analyses of the data, statistical analyses and the write up of the manuscript. AC, DF, PMV and JC have been involved in drafting the manuscript, interpretation of data and reviewed the manuscript. PMV and AC have been involved in statistical analyses. PV, GW, VM, JC and FP participated in the design and coordination of the study (Colaus Cohort) and assisted with the interpretation of the data and reviewed the manuscript. All authors read and approved the final manuscripts

## Pre-publication history

The pre-publication history for this paper can be accessed here:

http://www.biomedcentral.com/1471-2458/11/23/prepub
